# Optimization of an in vitro *Pseudomonas aeruginosa* Biofilm Model to Examine Antibiotic Pharmacodynamics at the Air-Liquid Interface

**DOI:** 10.1038/s41522-024-00483-y

**Published:** 2024-03-01

**Authors:** Xing Tan, Yanqin Huang, Amisha Rana, Nidhi Singh, Taylor C. Abbey, Hui Chen, Peter T. Toth, Zackery P. Bulman

**Affiliations:** 1 Department of Pharmacy Practice, University of Illinois Chicago College of Pharmacy, Chicago, IL USA; 2 Mass Spectrometry Core, Research Resources Center, University of Illinois Chicago, Chicago, IL USA; 3 Fluorescence Imaging Core, Research Resources Center, University of Illinois Chicago, Chicago, IL USA

**Keywords:** Biofilms, Antimicrobials

## Abstract

*Pseudomonas aeruginosa* is an important cause of lower respiratory tract infections, such as ventilator-associated bacterial pneumonia (VABP). Using inhaled antibiotics to treat VABP can achieve high drug concentrations at the infection site while minimizing systemic toxicities. Despite the theoretical advantages, clinical trials have failed to show a benefit for inhaled antibiotic therapy in treating VABP. A potential reason for this discordance is the presence of biofilm-embedded bacteria in lower respiratory tract infections. Drug selection and dosing are often based on data from bacteria grown planktonically. In the present study, an in vitro air-liquid interface pharmacokinetic/pharmacodynamic biofilm model was optimized to evaluate the activity of simulated epithelial lining fluid exposures of inhaled and intravenous doses of polymyxin B and tobramycin against two *P. aeruginosa* strains. Antibiotic activity was also determined against the *P. aeruginosa* strains grown planktonically. Our study revealed that inhaled antibiotic exposures were more active than their intravenous counterparts across biofilm and planktonic populations. Inhaled exposures of polymyxin B and tobramycin exhibited comparable activity against planktonic *P. aeruginosa*. Although inhaled polymyxin B exposures were initially more active against *P. aeruginosa* biofilms (through 6 h), tobramycin was more active by the end of the experiment (48 h). Together, these data slightly favor the use of inhaled tobramycin for VABP caused by biofilm-forming *P. aeruginosa* that are not resistant to either antibiotic. The optimized in vitro air-liquid interface pharmacokinetic/pharmacodynamic biofilm model may be beneficial for the development of novel anti-biofilm agents or to optimize antibiotic dosing for infections such as VABP.

## Introduction

Ventilator-associated bacterial pneumonia (VABP) is a common type of hospital-acquired infection and is associated with an unacceptably high all-cause mortality rate of 20–50%^1^. *P. aeruginosa* is the most common Gram-negative pathogen to cause VABP and has a propensity to form biofilms^2,3^. Recommended treatment of VABP infections includes antibiotics administered systemically that target the infecting pathogen^1^. However, it can be difficult to achieve adequate antibiotic concentrations at the site of infection in the lungs when using systemic antibiotics. Systemically administered antibiotics often used for the treatment of Gram-negative VABP infections, such as β-lactams, polymyxins, and aminoglycosides, do not fully penetrate the lung parenchyma^4–6^. In order to reach the site of infection (i.e. alveoli and epithelial lining fluid [ELF]), systemically administered antibiotics must cross the alveolar barrier of the capillary lumen, connective tissue, and alveolar epithelial cells, which contain various efflux pumps^7^. One potential approach to overcome the pharmacokinetic limitations of systemic drug delivery for infections in the lungs is through the use of inhaled antibiotics such as tobramycin and polymyxin B. Inhaled antibiotics can achieve high concentrations at the site of infection while reducing the systemic exposure and therefore, reduce the rates of many side effects. High concentrations of antibiotics detected following inhalation have also been previously shown to be active against planktonic bacteria in vitro^8,9^. However, this in vitro success has not consistently translated to patients, where inhaled antibiotics have often failed to improve clinical outcomes^10–13^. One potential explanation for this discordance is that the bacteria in VABP may consist of both planktonic and biofilm cells. The presence of both these communities was not fully considered in previous preclinical assessments of inhaled antibiotics.

Biofilms are thought to be a significant contributor to VABP pathogenesis and poor treatment outcomes, including for infections caused by *P. aeruginosa*^2,14,15^. Biofilms are composed of a complex multicellular bacterial community encased in an extracellular matrix (ECM) with variable oxygen gradients and metabolic states that contribute to their recalcitrance to antibiotics^16–19^. Biofilm formation on endotracheal tubes is correlated with the development of VABP^2^. Among patients with VABP, the causative bacteria exist in biofilms in 50–70% of cases^2,20,21^. It is believed that most patients with lung infections caused by *P. aeruginosa* have both planktonic and biofilm bacteria, with biofilms dispersed within secretions located near lung tissues or biofilms attached to the endotracheal tube^22–24^. Together, these data suggest biofilms play an important role in the development of VABP and its high rate of treatment failure^2,25^.

In contrast to planktonic bacteria, biofilms have several mechanisms to evade eradication by antibiotics. For example, the biofilm’s ECM can act as a physical barrier to antibiotic penetrance and the various metabolic and oxygen gradients within a biofilm can also impact bacterial growth and antibiotic activity^26,27^. Differences in the physical environment, such as air-liquid or liquid-liquid interfaces, can also influence biofilm growth and impact eradication^28^. Biofilms grown under liquid-liquid conditions can be more heterogenous, more susceptible to physical forces, and have weaker adhesion to their attached surface than those grown under air-liquid conditions if shear remains constant. These structural differences are associated with treatment differences; biofilms grown under air-liquid interfaces can be 100 times more resistant to antibiotics than those grown under liquid-liquid conditions^28,29^. Biofilms associated with respiratory tract infections, such as VABP, are thought to reside in an air-liquid interface. However, very few studies have assessed the pharmacodynamic activity of antibiotics against biofilms grown in the air-liquid interface^30^. Considering the unique properties of biofilms, especially those that are implicated in VABP, assessing simulated ELF concentrations against biofilm-embedded bacteria in a representative in vitro model may enable optimization of antibiotic treatment strategies for VABP.

The objectives of this study were to: 1) create a modified pharmacokinetic/pharmacodynamic (PK/PD) drip flow reactor and validate its ability to simulate human PK, and 2) compare the activity of inhaled and intravenous tobramycin and polymyxin B at simulated ELF concentrations against biofilm-embedded and planktonic *P. aeruginosa* cells.

## Results

### Pharmacokinetic validation of an air-liquid interface pharmacokinetic/pharmacodynamic biofilm model

**Fig. 1:**
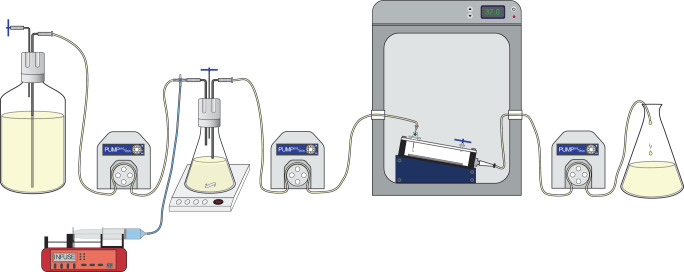
Diagram of the experimental setup for the air-liquid interface PK/PD biofilm model. In this diagram, media flows left to right with the aid of peristaltic pumps. The antibiotics are infused into an Erlenmeyer flask that has a fixed volume of growth media by an automated dosing pump (red) housed in a refrigerator (not shown). This flask is located on a magnetic stir plate to mix the antibiotics prior to being pumped into the incubator, where they continuously drip onto the biofilm. The biofilm is grown in a drip flow reactor, which has been previously described to grow the biofilm at the air-liquid interface. Waste media is then removed from the incubator and collected in a reservoir.

**Fig. 2:**
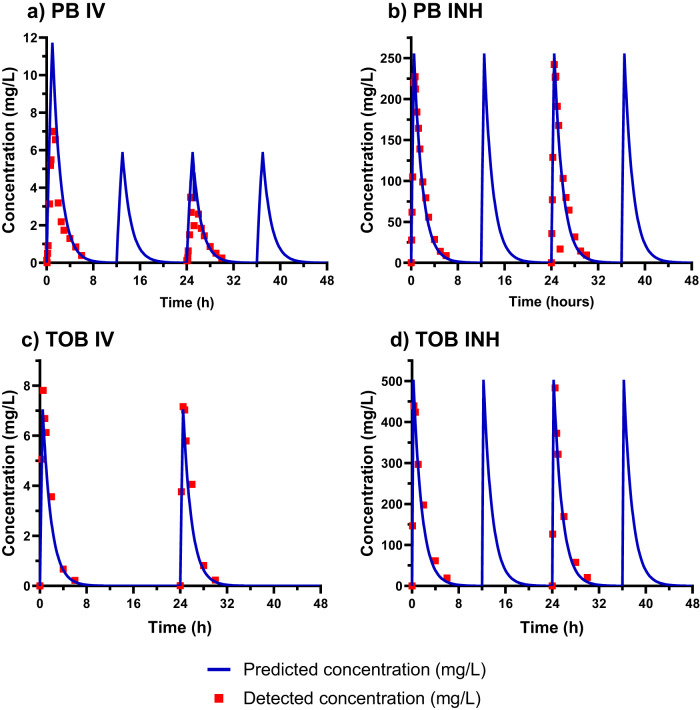
Pharmacokinetic data for antibiotics at simulated human concentrations for polymyxin B and tobramycin. Pharmacokinetics were validated for: **a** polymyxin B intravenous (PB IV), **b** polymyxin B inhaled (PB INH), **c** tobramycin intravenous (TOB IV), and **d** tobramycin inhaled (TOB INH), respectively. Blue lines and red squares represent predicted and detected concentrations, respectively.

We optimized an in vitro PK/PD biofilm model that exposes biofilms grown at the air-liquid interface with low shear to accommodate simulated human antibiotic exposures. *P. aeruginosa* biofilms were grown using the Drip Flow Biofilm Reactor (Model DFR 110-6PET, BioSurface Technologies Corp., Bozeman, MT), as previously described with some modifications to enable exposure to dynamic antibiotic concentrations (Fig. 1)^31^. The initial biofilm density in the Drip Flow Biofilm Reactor was very consistent between experiments with a mean ± SD of 9.45 ± 0.18 log_10_ CFU/cm^2^ and 9.13 ± 0.34 log_10_ CFU/cm^2^ for the PAO1 and AR0064 strains, respectively. The starting inoculum was also similar to previous studies using the Drip Flow Biofilm Reactor, which displayed an initial mean biofilm density of 9.29 log_10_ CFU/cm^2^^32^. There was a linear relationship between the predicted and detected pharmacokinetic profiles for polymyxin B intravenous (IV) (R^2^ = 0.91, slope = 0.62, and intercept = −0.05), polymyxin B inhaled (INH) (R^2^ 0.97, slope = 0.97, and intercept = 3.61), tobramycin IV (R^2^ = 0.97, slope = 1.11, and intercept = −0.18) and tobramycin INH (R^2^ = 0.97, slope = 1.01, and intercept = 7.80) (Fig. 2).

### Tobramycin and polymyxin B activity against biofilms

**Fig. 3:**
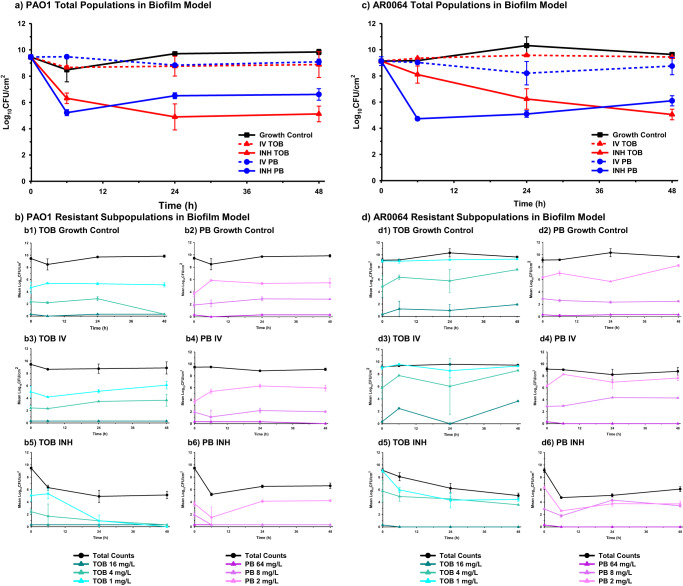
Pharmacodynamic activity of polymyxin B and tobramycin against *P. aeruginosa* in the air-liquid interface PK/PD biofilm model. The pharmacodynamic activity of polymyxin B (PB) and tobramycin (TOB) were assessed against (**a**) PAO1 and (**c**) AR0064 biofilms. Population analysis profiles tracked resistant subpopulations during antibiotic exposure for PAO1 (**b**) and AR0064 (**d**). Data are presented as the mean of duplicate runs +/− standard deviation.

Baseline PAO1 and AR0064 tobramycin MICs were 0.5 and 4 mg/L, respectively, and polymyxin B MICs were both 0.5 mg/L (Table 1). Tobramycin IV and polymyxin B IV were not active against biofilm-embedded PAO1 or AR0064 at any time point (Figs. 3 and 6). The activity of polymyxin B IV and tobramycin IV did not significantly differ at 6, 24, or 48 h (*P* > 0.05) (Supplementary Table 1). Against PAO1, tobramycin INH exhibited bactericidal activity as early as 6 h (3.14 ± 0.40 log_10_ CFU/cm^2^ reduction) and was maintained through 48 h (4.33 ± 0.60 log_10_ CFU/cm^2^ reduction). Polymyxin B INH was bactericidal at 6 h (4.23 ± 0.22 log_10_ CFU/cm^2^ reduction), however regrowth began to occur after that time point and this regimen was only microbiologically active at 24 h (2.95 ± 0.22 log_10_ CFU/cm^2^ reduction) and 48 h (2.85 ± 0.44 log_10_ CFU/cm^2^ reduction). Against AR0064, tobramycin INH displayed gradual killing. It was microbiologically active at 24 h (2.89 ± 0.78 log_10_ CFU/cm^2^ reduction) and bactericidal at 48 h (4.08 ± 0.41 log_10_ CFU/cm^2^ reduction). Polymyxin B INH was bactericidal at 6, 24, and 48 h (4.40 ± 0.12, 4.04 ± 0.25, and 3.03 ± 0.39 log_10_ CFU/cm^2^ reductions respectively). Polymyxin B INH was significantly more active than tobramycin IV at 6 h (*P* < 0.05) but tobramycin INH was significantly more active at 48 h (*P* < 0.05) (Supplementary Table 1). Tobramycin subpopulations capable of growing on tobramycin 4 mg/L plates emerged during exposure to tobramycin IV for PAO1 and AR0064 (Fig. 3). In contrast, tobramycin-resistant subpopulations did not emerge after tobramycin INH treatment for either isolate. Resistant subpopulations also did not emerge after polymyxin B treatment (IV or INH).

**Table 1:** Tobramycin and polymyxin B MICs at baseline and after 48 h of antibiotic treatment in air-liquid interface PK/PD biofilm model and planktonic one-compartment model

Bacterial Isolate	Tobramycin MICs, mg/L			
	Treatment Condition	Baseline MIC (0 h)	Post-Exposure MIC (48 h)	
			Biofilm	Planktonic
PAO1	Growth Control	0.5	1	1
	Tobramycin IV		1	1
	Tobramycin INH		0.5	1
AR0064	Growth Control	4	4	4
	Tobramycin IV		4	4
	Tobramycin INH		8	8

	Polymyxin B MICs, mg/L			
	Treatment Condition	Baseline MIC (0 h)	Post-Exposure MIC (48 h)	
			Biofilm	Planktonic
PAO1	Growth Control	1	2	1
	Polymyxin B IV		1	2
	Polymyxin B INH		1	1
AR0064	Growth Control	1	1	1
	Polymyxin B IV		2	2
	Polymyxin B INH		2	2

### Confocal laser scanning microscopy of biofilms

**Fig. 4:**
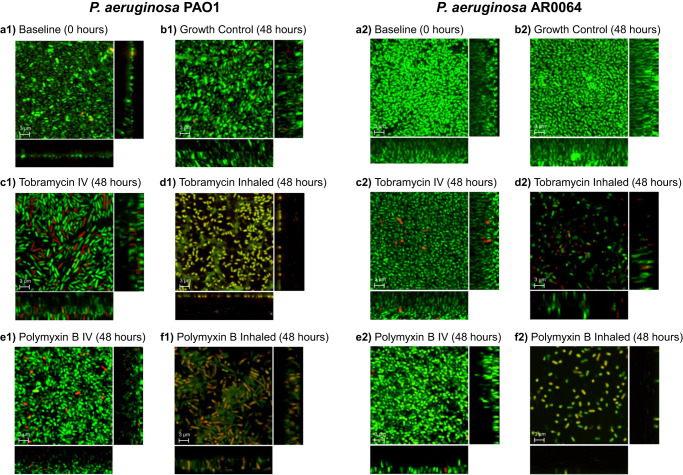
Confocal laser scanning microscopy with LIVE/DEAD stain of biofilm-embedded cells of *P. aeruginosa* before and after exposure to antibiotics. *P. aeruginosa* isolates (**A1-F1**) PAO1 and (**A2-F2**) AR0064 were imaged following exposure to polymyxin B and tobramycin. Representative images are shown at **a** 0 h, **b** 48 h of no treatment, **c** 48 h of simulated tobramycin intravenous treatment, **d** 48 h of simulated polymyxin B intravenous treatment, **e** 48 h of simulated tobramycin inhaled treatment, **f** 48 h of simulated polymyxin B inhaled treatment. Green indicates live cells stained by Syto 9, red or yellow cells indicate dead cells stained by propidium iodide. The central image is a compilation of all planes and the rectangle images on the right and below indicate XZ and YZ planes respectively to indicate the thickness of the biofilm. The average thickness of the imaged biofilm Z-stacks was 9.4 μm. Scale bars are 3 µm.

Confocal laser scanning microscopy (CLSM) was used to detect visual changes to the mature biofilms before and after antibiotic treatment (Fig. 4). Simulated tobramycin IV and polymyxin B IV treatment against both isolates resulted in more live cells than dead cells with minimal changes to spatial distribution or biofilm thickness compared to their respective growth control images at 48 h. The average thickness of the imaged biofilm Z-stacks was 9.4 µm. Exposure to tobramycin INH and polymyxin B INH resulted in decreased viability, altered spatial distribution, and decreased biofilm thickness for both PAO1- and AR0064-embedded biofilms. The tobramycin INH and polymyxin B INH regimens against PAO1 also appear to have disrupted the biofilm structure or biomass as seen by the green matrix of the biofilm dispersed between cells, which was not seen in AR0064.

### Tobramycin and polymyxin B activity against planktonic populations

**Fig. 5:**
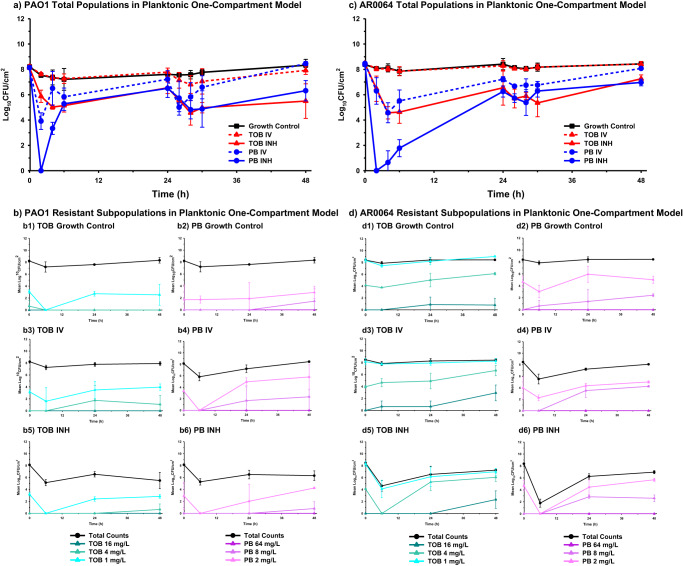
Pharmacodynamic activity of polymyxin B and tobramycin against *P. aeruginosa* in the planktonic one-compartment model. The pharmacodynamic activity of polymyxin B (PB) and tobramycin (TOB) were assessed against planktonic **a** PAO1 and **c** AR0064. Population analysis profiles tracked resistant subpopulations during antibiotic exposure for **b** PAO1 and **d** AR0064. Data are presented as the mean of duplicate runs +/− standard deviation.

**Fig. 6:**
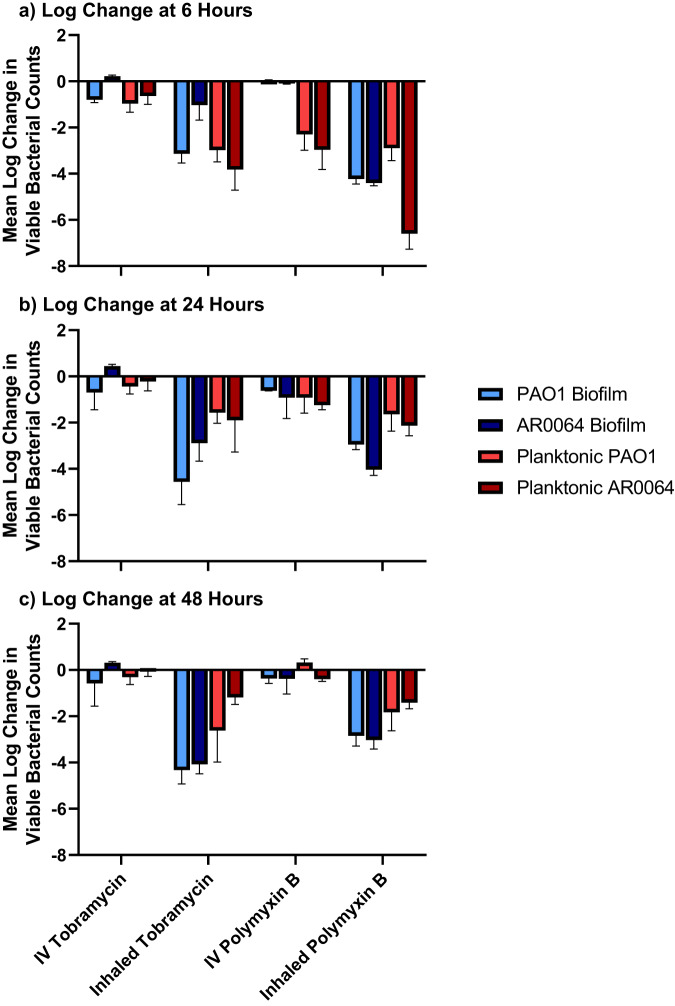
Summary of the pharmacodynamic activity of antibiotics against PAO1 and AR0064. Pharmacodynamic analyses were performed at **a** 6 h, **b** 24 h, and **c** 48 h. Activity from both the air-liquid interface PK/PD biofilm model (blue) and planktonic one-compartment model (red) are depicted with units of log_10_ CFU/cm^2^ and log_10_ CFU/mL, respectively. Data are presented as the mean log reduction. The T bars represent standard deviations.

Against planktonic cells, bacterial killing patterns were similar for both strains, aside from tobramycin INH, which caused greater killing of PAO1. Tobramycin IV was not bactericidal at any time point against either isolate and bacterial counts were similar to the growth control for most time points (Figs. 5 and 6). Polymyxin B IV caused ~4 log_10_ CFU/mL reduction between 2–4 h, but regrowth occurred by 48 h for both isolates. Polymyxin B IV was significantly more active than tobramycin IV at 6 and 24 h (*P* < 0.05) but there was no significant difference at 48 h (*P* > 0.05) (Supplementary Table 1). Polymyxin B INH reduced bacteria to an undetectable level at 2 h, however, regrowth was observed after 2 h. Final bacterial reductions were 1.83 ± 0.80 log_10_ CFU/mL and 1.41 ± 0.27 log_10_ CFU/mL at 48 h against PAO1 and AR0064, respectively. Similarly, tobramycin INH was bactericidal against both isolates at 4 h but regrowth was observed that resulted in final reductions of 2.62 ± 1.36 log_10_ CFU/mL and 1.19 ± 0.30 log_10_ CFU/mL at 48 h against PAO1 and AR0064, respectively. The activity of polymyxin B INH and tobramycin INH did not significantly differ at 6, 24, or 48 h (*P* > 0.05) (Supplementary Table 1). Following tobramycin exposure, an increase in subpopulations growing on tobramycin 4 mg/L plates was observed for isolate PAO1. For AR0064, there was an increase in the proportion of the population capable of growing on tobramycin 4 mg/L and 16 mg/L following tobramycin exposure (Fig. 5). Polymyxin B IV and INH led to increased growth of PAO1 on polymyxin B 2 mg/L plates relative to growth control. For AR0064, exposure to polymyxin B IV led to increased growth on 8 mg/L polymyxin B plates while polymyxin B INH only increased growth on 2 mg/L polymyxin B plates. After 48 h of antibiotic treatment in either the planktonic one-compartment model or biofilm model, both PAO1 and AR0064 isolates retained tobramycin and polymyxin B MICs within a single dilution of the baseline MIC (Table 1).

## Discussion

Inhaled antimicrobials are an attractive strategy to treat pneumonia because they can achieve high local concentrations with fewer toxicities than systemic antibiotics. However, recent randomized controlled trials for VABP have not conclusively demonstrated the efficacy of inhaled antibiotics^10–13^. There were several limitations to these clinical studies that makes their interpretation somewhat difficult including the use of a variety of inhalation devices and heterogeneous patient populations (e.g. various pathogens, resistance profiles, and uncontrolled concomitant intravenous antibiotic use). It is also important to note a trend toward benefit for inhaled antibiotics has been observed when considering microbiological response and for some antibiotic-resistant infections^33–35^. There is also a lack of consideration for the presence of biofilms in studies that have investigated the efficacy of inhaled antibiotics^36,37^. Planktonic and biofilm-embedded bacteria are often simultaneously present in acute pneumonias^15^. However, no previous studies have evaluated the activity of dynamic antibiotic concentrations against biofilms that mimic the environment of the lung at the air-liquid interface.

In this study, we optimized the air-liquid interface PK/PD biofilm model as a method to investigate concentrations of antibiotics that simulate human antibiotic exposures against biofilm-embedded bacteria. Previous studies that assessed dynamic antibiotic exposures against biofilms have primarily tested biofilms submerged in liquid growth media (e.g. CDC Biofilm Reactor), which may not be the most representative of biofilms found in the lungs. Considering biofilms’ growth characteristics depend on their environment, it is important to select an in vitro model that mimics the characteristics of the targeted infection^28,29,38^. Biofilms grown in an air-liquid environment produce biofilms that are thicker, less permeable, less susceptible to antibiotics, and have a smooth and homogenous surface morphology compared to biofilms grown at the liquid-liquid interface^28,29^. To model lung infections, the Drip Flow Biofilm Reactor model was selected to grow biofilms in an air-liquid interface under low shear, continuous nutrient flow conditions was selected^31^. Visualization of biofilm-embedded bacteria using CLSM following antibiotic treatment showed a decrease in viable bacteria, biofilm structure, and biofilm thickness that was particularly prominent for the inhaled regimens. These CLSM images correlated well with the viable counts and provided additional support that the drip flow reactor model can be used to assess antibiotic activity^39^.

In our air-liquid interface PK/PD biofilm model, inhaled polymyxin B initially exhibited a faster rate of killing compared to inhaled tobramycin (Fig. 6). However, by 48 h, killing by inhaled tobramycin was greater against each *P. aeruginosa* isolate. Tobramycin and the polymyxins can target different segments of the biofilm; tobramycin can kill the upper layer of more metabolically active cells whereas the polymyxins are active against deeper layers of the biofilm^40,41^. Tolerance to the polymyxins in the upper layer may be due to increased expression of *pmrAB* and *mexAB-oprM* whereas tolerance to aminoglycosides may be a result of reduced antibiotic uptake by the deeper metabolically inactive cells^42^. Thus, the enhanced activity of tobramycin may be due to a larger population of metabolically active cells in the biofilms. It is also possible that this outer layer of the biofilm thickens in the presence of polymyxin B, which could explain the regrowth pattern observed after 6 h of polymyxin B treatment. In contrast to the activity of inhaled antibiotics, intravenous tobramycin and polymyxin B regimens displayed limited activity, which suggests that this route of administration may not be preferred as monotherapy in patients with pneumonia caused by biofilm-forming *P. aeruginosa*. This is consistent with prior clinical studies that found intravenous tobramycin and polymyxin B to be associated with poor clinical outcomes in patients with pneumonia caused by *P. aeruginosa* and current treatment pneumonia treatment guidelines^1,43–46^.

Assessing the activity of the antibiotics against biofilm and planktonic populations confirmed that inhaled regimens were more active than intravenous regimens against *P. aeruginosa*. Intravenous tobramycin had no effect on the planktonic bacteria, which may in part be due to the tobramycin exposures being below the aminoglycoside *f*AUC/MIC PK/PD target value of >50 (Supplementary Table 2)^47^. In contrast, simulated intravenous polymyxin B had exposures in excess of the *f*AUC/MIC target for both isolates from 0–24 h (>30) but the exposure was less than this target between 24–48 h^48^. Consequently, polymyxin B displayed some initial activity against planktonic *P. aeruginosa* prior to regrowth between 24 and 48 h. Interestingly, inhaled tobramycin and polymyxin B had comparable activity against planktonic populations but tobramycin was more active after 48 h against the biofilms (Fig. 6). Taken together, these data may slightly favor the use of inhaled tobramycin over inhaled polymyxin B for treatment of infections in the lungs caused by biofilm-forming *P. aeruginosa*. This is consistent with a previous study conducted that found inhaled tobramycin to be more active than inhaled colistin for rats infected with *P. aeruginosa* and treated over 7 days^40^. In support of the validity of our models, killing by the inhaled polymyxin B dose across both the biofilm and planktonic models in this study (~1.6–3 log_10_ 24 h kill) was similar to the ~2 log_10_ kill previously observed for a comparable aerosolized polymyxin B dose administered to mice infected with *P. aeruginosa* PAO1^49^. Although less active at 48 h, inhaled polymyxin B was more active than inhaled tobramycin at 6 h and may still be a valuable alternative to treat these infections. The two *P. aeruginosa* strains largely displayed similar responses to tobramycin and polymyxin B. However, it is interesting to note that PAO1 was observed to regrow faster, despite having the same MIC, following the first inhaled polymyxin B dose in both models. The mechanism for this difference warrants additional testing. It is also important to note that the experiments in the present study were only conducted for 48 h, which is a shorter duration than most patients are treated with antibiotics for VABP. Additional experiments are required to determine whether the air-liquid interface PK/PD biofilm model can reproducibly sustain biofilm growth beyond 48 h and if the activity of antibiotics varies between 48 h and later time points.

Remarkably, the activity of tobramycin and polymyxin B were lower against planktonic *P. aeruginosa* populations starting at 24 h. Following antibiotic treatment, the MICs remained unchanged from baseline and heteroresistant subpopulations did not appreciably change, suggesting the mechanism for the lower activity against planktonic cells was not likely due to the emergence of resistance in this population. One potential explanation is that the antibiotics in the planktonic one-compartment models selected for a subpopulation of slow-growing or stationary phase cells that were antibiotic-tolerant. Indeed, previous studies have shown that planktonic *P. aeruginosa* in stationary phase can be more recalcitrant to antibiotics than *P. aeruginosa* in biofilms, including tobramycin^50^. It is also possible that the 10× higher concentration of TSB needed to support bacterial growth in the one-compartment model versus the TSB concentration required for the biofilm model enabled the planktonic *P. aeruginosa* to regrow more quickly following initial antibiotic exposure (Supplementary Fig. 1). The surprising finding that antibiotics were more active overall against biofilms than planktonic cells in our models warrants additional investigation to fully explain the mechanism. The fact that neither inhaled polymyxin B nor inhaled tobramycin were able to eradicate *P. aeruginosa* populations supports the potential need for treatment with antibiotic combinations^39,51,52^.

There are a few limitations to this study. First, both of the in vitro models lacked an immune system, which is a limitation inherent to all in vitro models. Thus, total bacterial killing observed in the present study may underestimate bacterial killing anticipated for these antibiotics in an immunocompetent host. Second, the drip flow reactor model has been shown to occasionally promote growth of heterogeneous biofilms. However, the replicates in our experiments had notably small standard deviations in viable bacterial counts at each time point that were also consistent with previous experiments^32^, suggesting a high degree of reproducibility. It is also important to acknowledge that in this in vitro study the antibiotics for the inhaled regimens were not aerosolized during administration as they would be in patients. Although the pharmacokinetic profiles we tested are representative of the antibiotic concentrations that may be expected in patients following inhaled administration, this study does not address the challenges associated with delivering inhaled antibiotics uniformly to the distal airway. There is a great deal of variability in the pharmacokinetics of drugs in the ELF following inhaled administration due to patient and formulation-related factors, among others^7,53^. Optimization of the drug formulation and delivery method for inhaled administration remain an important area for continued research and are critical to safely deliver high antibiotic concentrations to the pathogens in the lungs. Nonetheless, we anticipate that if the antibiotic concentrations explored in the present study can be achieved through optimal inhaled administration in the lungs of patients with VABP, the pharmacodynamic activity we observed will remain relevant.

In conclusion, the air-liquid interface PK/PD biofilm model was optimized for testing human-simulated antibiotic doses against biofilms growing in an environment that may be more consistent with lung infections such as VABP. The air-liquid interface PK/PD biofilm model may be useful to improve development of novel anti-biofilm agents, inhaled antimicrobial agents, or to optimize currently available inhaled antimicrobial agents. Surprisingly, the tobramycin and polymyxin B regimens were generally more active against the *P. aeruginosa* biofilms than they were against planktonic cells, which may be due to the development of a stationary phase population that was antibiotic tolerant. Inhaled polymyxin B was initially more active than inhaled tobramycin (through 6 h) against *P. aeruginosa* biofilms, but by the end of the experiment (48 h) tobramycin was more active. This suggests that inhaled tobramycin may be slightly preferred over inhaled polymyxin B for treating pneumonia caused by *P. aeruginosa* isolates that are not resistant to either agent.

## Methods

### Bacterial strains and susceptibility testing

*Pseudomonas aeruginosa* reference strain PAO1 and clinical isolate *P. aeruginosa* AR Bank #0064 (AR0064) were selected based on their potential to form biofilms in the drip flow reactor and their susceptibility to tobramycin and polymyxin B (Table 1)^54^. PAO1 is carbapenem susceptible (meropenem MIC is 1 mg/L) and AR0064 is carbapenem resistant (meropenem MIC ≥ 8 mg/L).

Tobramycin (AK Scientific, CA) and polymyxin B (Sigma-Aldrich, MO) were used and fresh antibiotic stocks were prepared on the day of each experiment. Broth microdilution was performed in triplicate in CAMHB to determine MICs at baseline and after 48 h of growth in both the biofilm and one-compartment models, according to CLSI^55^. MICs performed after antibiotic exposure in the biofilm and one-compartment models were passaged one time on tryptic soy agar (TSA) prior to performing MICs. Modal MICs were reported.

### Simulated antibiotic dosing regimens

Human ELF concentration-time profiles of tobramycin and polymyxin B, two agents commonly used for respiratory tract infections due to *P. aeruginosa*, were simulated in this study^1^. Tryptic soy broth (TSB) (BD, Sparks) 30 g/L was used in the one-compartment model. TSB 3 g/L minimal media was used in the dynamic biofilm model to facilitate biofilm formation^31^. Nutrient concentration of 3 g/L was internally optimized for biofilm growth in the drip flow reactor model; lower concentrations did not facilitate growth and higher concentrations yielded similar or lower biofilm density. Tobramycin IV exposures were based on a PK study in patients with pneumonia^56^. Simulated tobramycin INH exposures were based on PK studies in critically ill and cystic fibrosis patients^57–61^. Polymyxin B concentrations after IV administration were extrapolated from critically ill patients with VABP being treated with colistin and scaled according to recommended polymyxin B dosing^62,63^. Simulated polymyxin B INH exposures were based on PK data from critically ill patients with VABP after accounting for the bound fraction^62,64^. Protein binding in ELF was assumed to be approximately 50% for all regimens^64–67^. Antibiotics were dissolved in normal saline and administered by syringe pumps (NE-1000 × 2; New Era Pumps Systems).

Simulated antibiotic regimens and predicted PK parameters:

1. Tobramycin IV: 7–10 mg/kg IV over 0.5 h every 24 h
  1. *f*C_max, ELF_: 7 mg/L
  2. t_1/2__,__ELF_: 1 h
  3. *f*AUC_0-24_: 12.00 mg·h/L
2. Tobramycin INH: 300 mg inhaled over 0.25 h every 12 h
  1. *f*C_max, ELF_: 500 mg/L
  2. t_1/2__,__ELF_: 1 h
  3. *f*AUC_0-24_: 1586.57 mg·h/L
3. Polymyxin B IV:
  1. Loading dose: 2–2.5 mg/kg IV over 1 h × 1 dose
    *f*C_max, ELF_: 11.68 mg/L
    t_1/2__,__ELF_: 1 h
  2. Maintenance dose: 1.25 mg/kg IV over 1 h every 12 h, starting 12 h after the loading dose
    *f*C_max, ELF_: 5.84 mg/L
    t_1/2,__ELF_: 1 h
  3. *f*AUC_0-24_: 35.24 mg·h/L
  4. *f*AUC_24-48_: 23.51 mg·h/L
4. Polymyxin B INH: 300 mg inhaled over 0.5 h every 12 h
  1. *f*C_max, ELF_: 250 mg/L
  2. t_1/2__,__ELF_: 1 h
  3. *f*AUC_0-24_: 874.45 mg·h/L

### Pharmacokinetic analysis with LC-MS/MS

Antibiotic samples in TSB were obtained from the PK/PD models to validate predicted concentrations. Polymyxin B samples in TSB (100 μL) were prepared by mixing with 20 µL of 1.0 M NaOH, followed by deproteinization with acetonitrile containing 1% formic acid. The supernatant after centrifugation was mixed with the internal standard working solution (2 ug/mL colistin sulfate) for LC/MS analysis. Tobramycin samples were prepared by diluting 5×, 100×, 250×, 625×, or 2500×, depending on the concentration, in 50% MeOH containing 0.1% formic acid to get to the linear range of the curve for detection. The diluted samples were mixed with the internal standard working solution (10 ng/mL Lysergic acid diethylamide-d3).

The samples were analyzed by LC-MS/MS (QTRAP 6500 [AB Sciex, Framingham, MA] coupled with Agilent 1290 UPLC system [Agilent, New Castle County, DE]). The separation was conducted using an Acquity UPLC BEH C18 column (50_2.1 mm, 1.7 μm; Waters, Milford, MA) for polymyxin B and Poroshell 120 EC-C18 column (100_2.1 mm, 2.7 μm, Agilent, New Castle County, DE) for tobramycin with 0.1% formic acid in H_2_O as mobile phase A and 0.1% formic acid in acetonitrile as mobile phase B. Mass spectrometry data was acquired by multiple reaction monitoring scan in positive mode. The electrospray ionization voltage and source temperature were kept at 4.5 kV and 500 °C. The analytes and internal standards were measured by monitoring their transitions to signature product ions. The data analysis was conducted by Sciex MultiQuant software (Version 3.0.3, AB Sciex, Framingham, MA, USA). The accuracy of the tobramycin assay was 97.2% and the coefficient of variation (CV%) was 6.2%. The accuracy and CV% for the polymyxin B assay were 100.4% and 4.1%, respectively.

### Air-liquid interface pharmacokinetic/pharmacodynamic biofilm model

*P. aeruginosa* biofilms grown in the Drip Flow Biofilm Reactor were exposed to dynamic antibiotic concentrations (Fig. 1). An intermediate vessel between the media carboys and the drip flow reactor was added to accommodate antibiotic dosing and clearance. Fresh broth is continuously pumped into and out of the intermediate vessels at the same flow rate by peristaltic pumps (Masterflex L/S series, Cole-Parmer, US). A magnetic stir bar in the intermediate vessels was used to ensure homogeneity. Media leaving the intermediate vessel, with or without antibiotics, is pumped into the drip flow reactor, where it runs across the biofilm before leaving the chamber through an effluent port and being pumped into a waste flask another peristaltic pump. Antibiotics are administered into the intermediate vessels using syringe pumps (NE-1000 × 2; New Era Pump Systems Inc.).

Biofilms were grown as previous described by inoculating each reactor chamber with of 15 mL of ~6 × 10^6^ CFU/mL *P. aeruginosa* grown in 3 g/L TSB^31^. Chambers were incubated at 37 °C without media flowing for 6 h while the reactor laid flat. Biofilms were then formed on glass coupons with a surface area of 19.35 cm^2^ (2.54 cm × 7.62 cm) secured in each of the reactor chambers under low-shear conditions by initiating continuous media flow at 0.8 mL/min/chamber with 3 g/L TSB. Biofilm growth continued for 48 h with the reactor at a 10˚ angle to allow for media outflow to waste containers. After 48 h of biofilm formation, experiments were initiated and antibiotic dosing began (time = 0 h). The media flow rate was maintained at 0.8 mL/min/chamber throughout the experiment to preserve the integrity of the biofilm. Biofilm sampling was done at 0 h (mature biofilms prior to antibiotic exposure), 6 h (mature biofilms exposed to antibiotics for 6 h), 24 h (mature biofilms exposed to antibiotics for 24 h), and 48 h (mature biofilms exposed to antibiotics for 48 h). At each time point, the glass slide was removed from the reactor, rinsed in 0.9% normal saline twice to remove planktonic cells, and then scraped into 50 mL of 0.9% normal saline using a rubber policeman. Each sample was vortexed for 1 min, then biofilms were disaggregated using a homogenizer (Labgen-125;115VAC, Cole-Parmer, US), as previously described^31^. Biofilm-embedded viable bacterial cells were then enumerated by serial dilution and viable colony counting. Experiments were performed in duplicate. The log density (CFU/cm^2^) of one coupon was calculated as previously described^31^.

### One-compartment pharmacokinetic/pharmacodynamic model

The one-compartment pharmacokinetic/pharmacodynamic model was used to evaluate the microbiological response of simulated human drug exposures against the *P. aeruginosa* isolates grown in the planktonic phase over 48 h. The four-drug regimens were simulated and tested against each isolate. Experiments were conducted as we previously described^68^. Briefly, TSB 30 g/L was continuously pumped into central reservoirs containing 105 mL of a starting bacterial inoculum at ~10^8^ CFU/mL. Waste media was removed at the same rate fresh media was infused into the central reservoir to ensure it maintained a consistent volume. The central reservoirs were placed in a 37 °C incubator and mixed constantly by a magnetic stir bar to ensure homogeneity. Drugs were infused into the central reservoir via syringe pumps (NE-1000 × 2; New Era Pumps Systems). Viable bacterial cell counts were determined at 0, 2, 4, 6, 24, 26, 28, 30 and 48 h after drug administration. Experiments were performed in duplicate.

To compare the activity of tobramycin and polymyxin B in the planktonic and biofilm PK/PD models, two-sided student’s t-tests were used and a *P* value of <0.05 was considered statistically significant. Tobramycin IV vs. polymyxin B IV and tobramycin INH vs. polymyxin B INH were compared at 6, 24, and 48 h by combining data for both strains. Thus, data in each group were based on at least 4 biological replicates (2 per strain). Statistical analyses were performed using GraphPad Prism 10.1.1.

### Population analysis profiles

To assess the emergence of subpopulations with resistance against tobramycin or polymyxin B, population analysis profiles (PAPs) were performed. Bacteria from planktonic and biofilm PK/PD models were plated onto antibiotic embedded TSA (tobramycin 1 mg/L, 4 mg/L, and 16 mg/L and polymyxin B 2 mg/L, 8 mg/L, and 64 mg/L) at 0 h, 6 h, 24 h, and 48 h after antibiotic exposure. Antibiotic concentrations were selected based on clinical breakpoints^69^. Plates were incubated at 37 °C for ~48 h prior to counting.

### Pharmacodynamic analysis

Response to antibiotics were described by a change in mean log_10_ CFU/cm^2^ or log_10_ CFU/mL for biofilm-embedded cells or planktonic cells, respectively. Bactericidal activity in both the dynamic biofilm model and one-compartment model was defined as ≥3 log_10_ reduction (CFU/cm^2^ or CFU/mL, respectively) at each sampling time point compared to 0 h as previously described^39,51^. Microbiologic activity in the dynamic biofilm model was defined as ≥1 log_10_ CFU/cm^2^ at each sampling time point compared to 0 h^39,51^. Mean (±SD) log_10_ reductions are reported.

### Confocal laser scanning microscopy of biofilms

The visual qualitative effect of different antibiotic regimens on viability (live or dead cells), shape, size, spatial distribution, and thickness of the biofilm was assessed by confocal laser scanning microscopy (CLSM). Fully formed biofilms on coupons at baseline (0 h) or following antibiotic treatment (48 h) in the air-liquid interface PK/PD biofilm model were rinsed with 0.9% normal saline stained with SYTO 9/propidium iodide (LIVE/DEAD BacLight Bacterial Viability Kit, ThermoFisher Scientific, USA), and rinsed again with 0.9% normal saline to remove excess stain^31^. SYTO 9 is a green-fluorescent stain that is cell permeable that indicates live cells, while propidium iodide is a red-fluorescent nuclear and chromosome counterstain that indicates dead cells. Coupons were observed with CLSM using a Zeiss LSM 710 META confocal microscope (Carl Zeiss Microscopy, GmbH, Germany) and software Zen v2.3, equipped with a 488 nm argon laser and 561 nm DPSS laser using alpha-PlanApo 63× oil immersion objective (1.45 NA). Images were obtained in four random fields on each coupon. With a 4× zoom factor we imaged a 34 μm × 34 μm area. The number of Z-planes were kept at an average of 314 slices (9.4 μm full Z-stack height), at which Z-stack height the thickest biofilm was fully imaged through and made comparisons possible. Images were processed with Imaris software (Bitplane AG, Switzerland).

### Static Concentration Time-Kill Assays

Static concentration time-kill assays (SCTKAs) were used to assess if the difference in TSB concentration between the one-compartment and biofilm models may have contributed to the lower antibiotic activity observed for planktonic cells compared to biofilm cells (Figs. 3 and 5). SCTKAs were performed as previously described using PAO1 at a starting inoculum of ~10^8^ CFU/mL. Polymyxin B (2.5 mg/L) and tobramycin (5 mg/L) were tested at clinically relevant concentrations. PAO1 was prepared in TSB at a broth concentration of 3 g/L or 30 g/L and brought to log phase prior to addition of antibiotics. Aliquots were removed after 0, 2, 4, 6, 24, 26, 26, 30, and 48 h for serial dilution and plating to quantify viable colonies at each time point.

### Reporting summary

Further information on research design is available in the Nature Research Reporting Summary linked to this article.

### Supplementary information

Supplemental Material

Reporting Summary

## Data Availability

Data supporting the findings of this study are available from the corresponding author upon request.
