# Supplementary material for: Optimization of an in vitro Pseudomonas aeruginosa Biofilm Model to Examine Antibiotic Pharmacodynamics at the Air-Liquid Interface
Source: NPJ Biofilms Microbiomes. 2024 Mar 1;10:16. doi: 10.1038/s41522-024-00483-y (PMC10907394; doi:10.1038/s41522-024-00483-y)
Supplement: Supplementary file 1 — Supplemental Material [file 41522_2024_483_MOESM1_ESM.pdf]

SUPPLEMENTARY INFORMATION

Supplementary Figure 1. Activity of polymyxin B (**A**) and tobramycin (**B**) against PAO1 at the TSB concentrations that were used in the dynamic one-compartment model (30 g/L) and the air-liquid interface PK/PD biofilm model (3 g/L). When PAO1 was grown in the media with a lower concentration of TSB, polymyxin B displayed enhanced activity at 2 and 4 hours while tobramycin displayed enhanced activity between 2 and 48 hours.

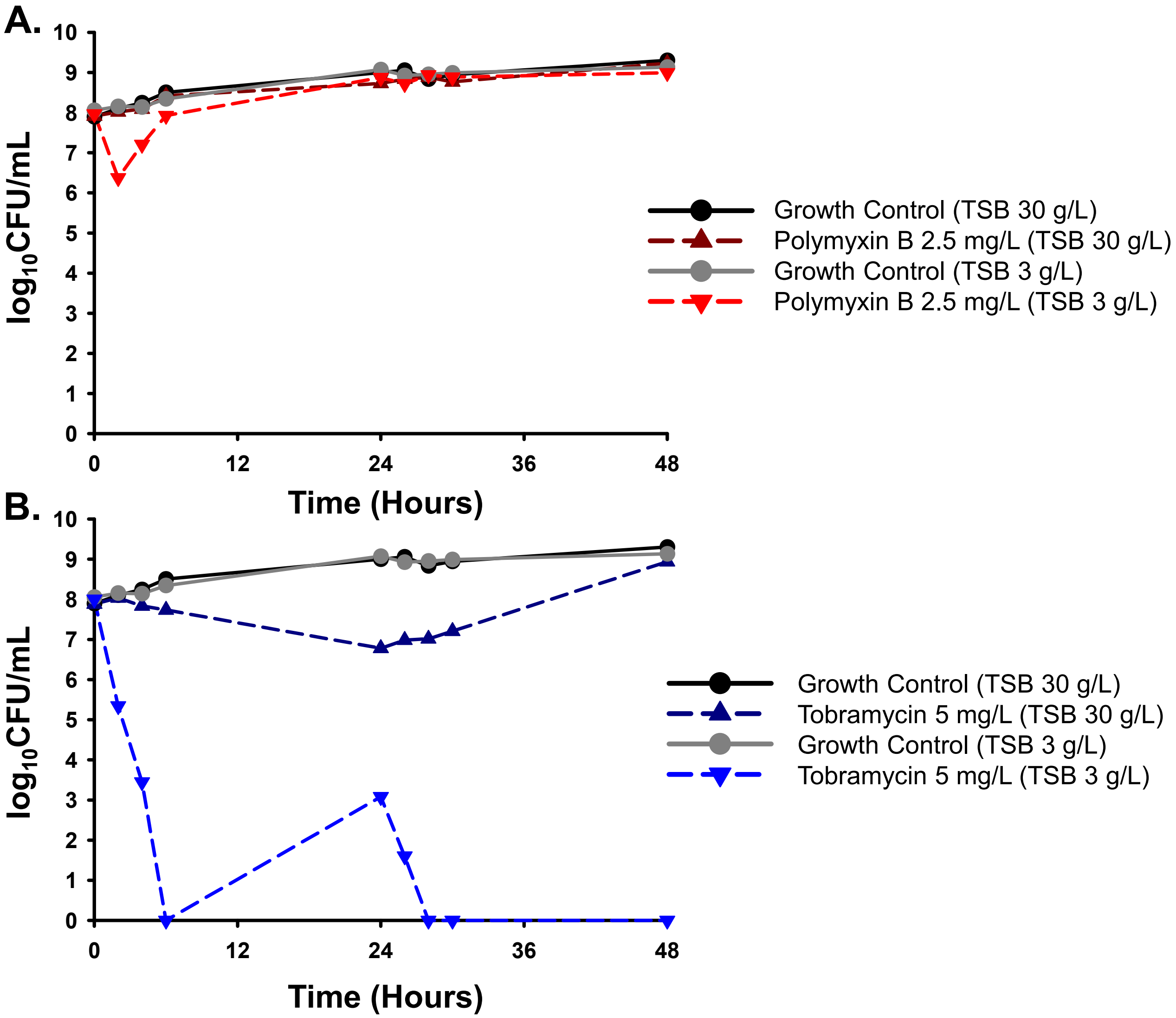

Supplementary Table 1. Analyses of the mean viable counts of *P. aeruginosa* following exposure to tobramycin and polymyxin B in the biofilm and planktonic PK/PD models. Data were combined from both strains (PAO1 and AR0064) to calculate the mean values. Two-sided student's t-tests were used to compare tobramycin to polymyxin B at 6, 24, and 48 hours for each of the simulated modes of administration (intravenous and inhaled). A *P* value of <0.05 was considered statistically significant.

**Biofilm: 6 Hours**				
**Treatment Regimen**	**Mean Log_{10}CFU/mL**	***P* value**	**t**	**df**
Tobramycin IV (n=4)	9.003	0.346	1.022	6
Polymyxin B IV (n=4)	9.249			
Tobramycin INH (n=4)	7.206	**0.009**	3.824	6
Polymyxin B INH (n=4)	4.974			
**Biofilm: 24 Hours**				
**Treatment Regimen**	**Mean Log_{10}CFU/mL**	***P* value**	**t**	**df**
Tobramycin IV (n=4)	9.166	0.201	1.436	6
Polymyxin B IV (n=4)	8.518			
Tobramycin INH (n=4)	5.566	0.890	0.144	7
Polymyxin B INH (n=5)	5.656			
**Biofilm: 48 Hours**				
**Treatment Regimen**	**Mean Log_{10}CFU/mL**	***P* value**	**t**	**df**
Tobramycin IV (n=4)	9.158	0.565	0.610	6
Polymyxin B IV (n=4)	8.917			
Tobramycin INH (n=4)	5.087	**0.006**	4.120	6
Polymyxin B INH (n=4)	6.352			
**Planktonic: 6 Hours**				
**Treatment Regimen**	**Mean Log_{10}CFU/mL**	***P* value**	**t**	**df**
Tobramycin IV (n=4)	7.560	**0.003**	4.697	6
Polymyxin B IV (n=4)	5.672			
Tobramycin INH (n=4)	4.885	0.262	1.239	6
Polymyxin B INH (n=4)	3.531			
**Planktonic: 24 Hours**				
**Treatment Regimen**	**Mean Log_{10}CFU/mL**	***P* value**	**t**	**df**
Tobramycin IV (n=4)	8.028	**0.032**	2.792	6
Polymyxin B IV (n=4)	7.221			
Tobramycin INH (n=4)	6.546	0.743	0.343	6
Polymyxin B INH (n=4)	6.379			
**Planktonic: 48 Hours**				
**Treatment Regimen**	**Mean Log_{10}CFU/mL**	***P* value**	**t**	**df**
Tobramycin IV (n=4)	8.169	0.676	0.439	6
Polymyxin B IV (n=4)	8.265			
Tobramycin INH (n=4)	6.376	0.724	0.370	6
Polymyxin B INH (n=4)	6.641			

***P* values < 0.05 appear in bold**

Supplementary Table 2. Predicted pharmacokinetic/pharmacodynamic exposures for tobramycin and polymyxin B against *P. aeruginosa* PAO1 and AR0064. Aminoglycoside activity has been correlated with *f*AUC/MIC and fC_{max}/MIC whereas the activity of polymyxin B is primarily attributed to optimizing the *f*AUC/MIC.

**Bacterial Isolate**	**Tobramycin IV**		**Tobramycin INH**		**Polymyxin B IV**	**Polymyxin B INH**
	*f*AUC/MIC	fC_{max}/MIC	*f*AUC/MIC	fC_{max}/MIC	*f*AUC/MIC	*f*AUC/MIC
PAO1	24	14	3173.1	1000	35.2 / 23.5[1]	874.5
AR0064	3	1.8	396.6	125	35.2 / 23.5[1]	874.5

[1]The first value is the *f*AUC/MIC ratio from 0-24 hours while the second value is the *f*AUC/MIC ratio from 24-48 hours.
